# Graph learning based suicidal ideation detection via tree-drawing test

**DOI:** 10.3389/fpsyt.2025.1617650

**Published:** 2025-07-18

**Authors:** Ye Liu, Jiashuo Zheng, Yang Zeng, Fang Luo, Xuetao Tian

**Affiliations:** ^1^ School of Future Technology, South China University of Technology, Guangzhou, China; ^2^ Faculty of Psychology, Beijing Normal University, Beijing, China; ^3^ Engineering Research Center of Integration and Application of Digital Learning Technology, Ministry of Education, Beijing, China

**Keywords:** suicidal ideation detection, tree-drawing test, projective test, graph learning, graph convolutional network

## Abstract

**Introduction:**

Adolescent suicide is a critical public health concern worldwide, necessitating effective methods for early detection of high suicidal ideation. Traditional detection methods, such as self-report scales, suffer from limited accuracy and are susceptible to personal concealment. Automatic methods based on artificial intelligence techniques are more accurate, while they often lack scalability due to strict data requirements. In order to achieve a balance between accuracy and scalability, this paper introduces the Tree-Drawing Test (TDT) as an effective tool for suicidal ideation detection, and proposes a novel graph learning approach to enable its automatic application.

**Methods:**

The proposed method first constructs a semantic graph based on psychological features annotated automatically from tree-drawing images, and leverages a Graph Convolutional Network (GCN) model to realize individual suicidal ideation detection. To evaluate this method, a real dataset of 806 students from primary and secondary school in Shaanxi Province, China, is collected, and some metrics including macro-F1, G-mean, and false positive rate are used.

**Results:**

The results demonstrate that the proposed method significantly outperforms traditional machine learning and convolution neural network approaches. The ablation study demonstrates the effectiveness of feature “leaves and fruits” in detecting suicidal ideation. Further experiments demonstrate that the proposed method remains stable even when the model is disturbed, such as when a tree-drawing image cannot be fully represented.

**Discussion:**

The proposed method highlights its effectiveness in large-scale suicidal ideation screening, as it not only achieves high detection performance but also maintains model stability while remaining flexible and adaptable.

## 1 Introduction

Adolescent suicide has been a public health concern. Data from the World Health Organization (WHO) shows that more than 1.5 million adolescents and young adults aged 10 to 24 years died in 2021 (1), and suicide has been the fourth leading cause of death among young people from the ages of 15 to 29 (2). Meanwhile, approximately one-third of adolescents experiencing suicidal ideation progress to developing suicide plans, and approximately 60% of those have attempted suicide (3). In this case, early detection and intervention of suicidal ideation through large-scale screening is crucial to promoting healthy adolescent development.

**Figure 1:**
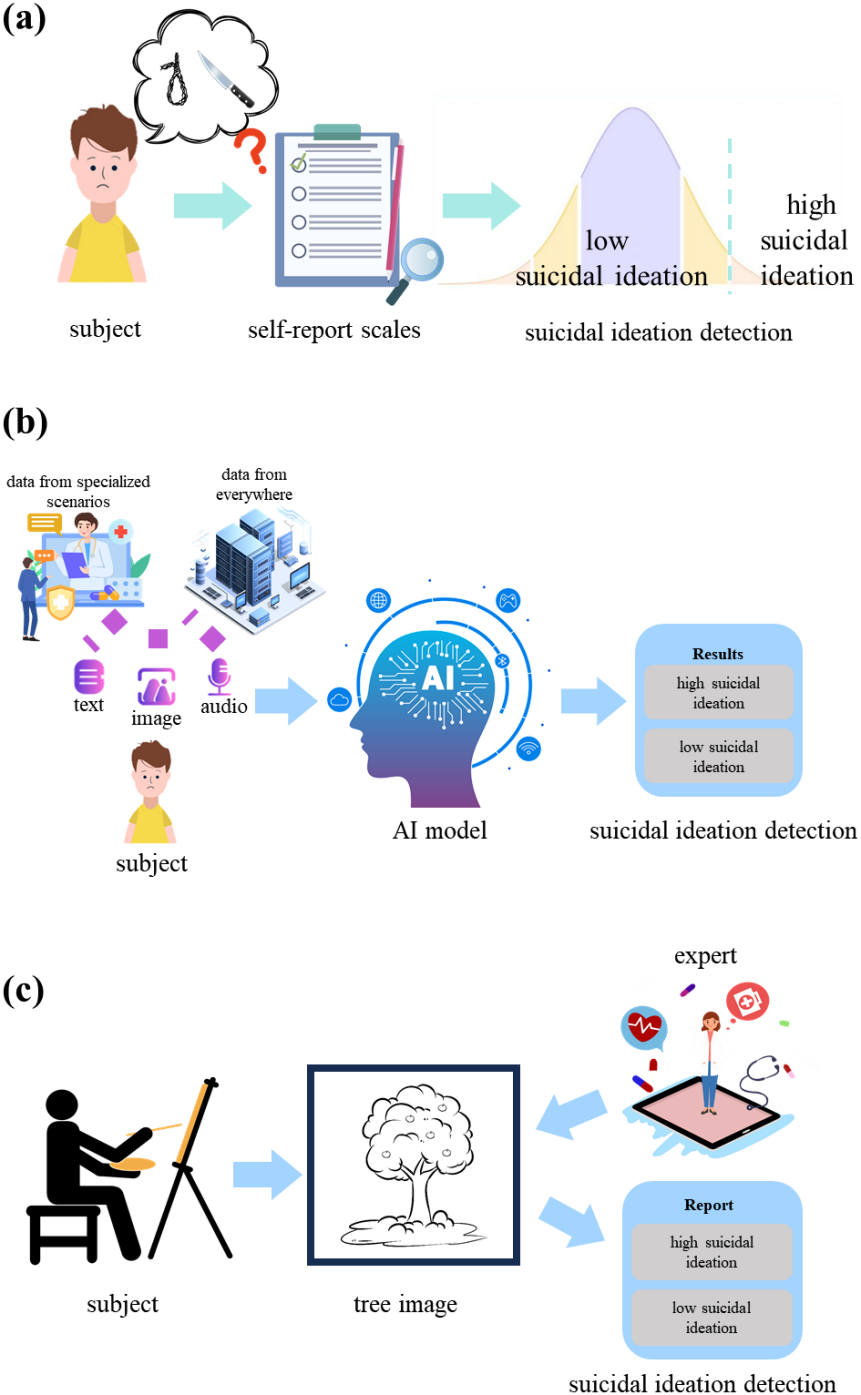
Flowchart of suicidal ideation detection. **(a)** Flowchart of suicidal ideation detection via self-report scales. **(b)** Flowchart of suicidal ideation detection via automatic AI model. **(c)** Flowchart of suicidal ideation detection via the drawing projective tests.

Current tools for detecting suicidal ideation mainly contain self-report scales, artificial intelligence (AI)-driven detection methods and projective tests as shown in **Figure 1**. Although self-report scales [e.g., Beck Scale (4)] facilitate efficient large-scale screening for suicidal ideation, their results are susceptible to inaccuracies and self-presentation biases, particularly those arising from intentional self-concealment of sensitive information. AI-driven detection methods, which utilize multi-modal data such as text (5–7), audio (8–10), video (11, 12), electroencephalogram (EEG) (13, 14), etc., demonstrate superior accuracy but lower scalability, as they require specialized data collection process. For instance, researchers collected video and audio data from clinical interviews to analyze smile and gaze behavior, and used SVM method to classify suicidal patients, psychiatric patients and control groups (11). Another study collected resting-state fMRI data from depressed patients in clinical suicidal crisis, constructed a feature mask via two-sample *t*-test on regional connectivity, and designed a semisupervised clustering framework using the mask for suicidal ideation prediction (15). Projective tests like the Tree-Drawing Test (TDT) have been applied to identify individuals with psychological states like depressive disorders (16) and dissociative identity disorder (17). By leveraging ambiguous stimuli to uncover latent emotions, these methods mitigate subjective bias, enhance response authenticity, and facilitate the scalability of psychological evaluation. However, this approach heavily relies on expert interpretation and cannot achieve a timely diagnosis.

To address this challenge of supporting large-scale screening, computer vision methods, such as image processing and Convolutional Neural Network (CNN), have been applied to the TDT successfully (18–20). However, since they focus more on the texture features of tree drawing images and lack the prior knowledge of psychology, it is difficult for them to capture the highly relevant characteristics between TDT and psychological states. Against this background, this paper aims to leverage graph learning methods to employ explainable semantic features related to suicidal ideation and achieve powerful detection capabilities simultaneously, thereby promote TDT’s application in the field of suicidal ideation detection.

## 2 Materials and methods

### 2.1 Dataset

**Figure 2:**
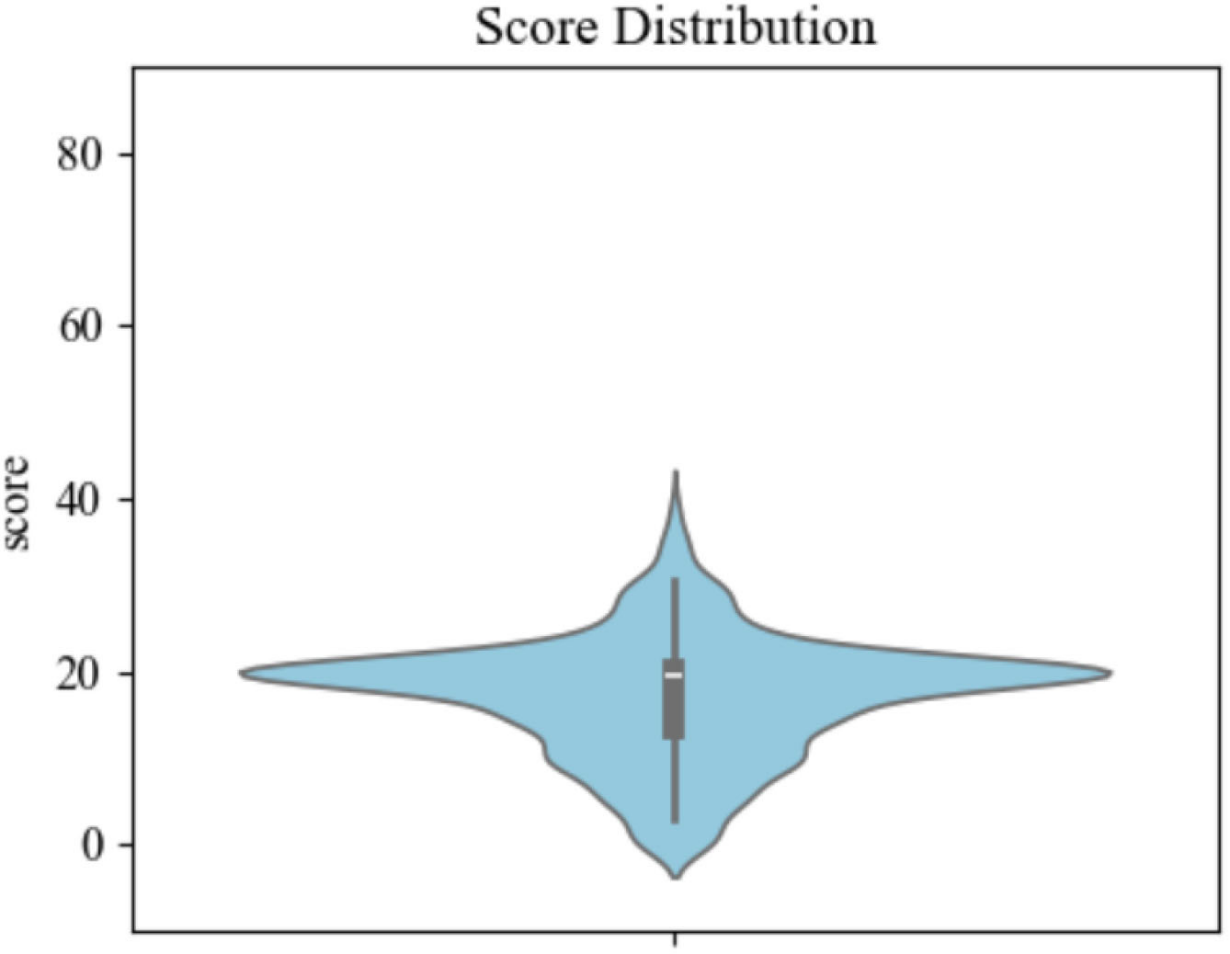
The score distribution from the self-report scale of suicidal ideation.

To conduct this research, we established a dataset involving lots of students from primary and secondary schools in Shaanxi Province, China. There are totally 806 participants (392 males and 414 females). Each participant completed the Beck Scale for Suicide Ideation and drew a tree on A4-sized white paper as part of the Tree-Drawing Test. To ensure the effectiveness of scale, we evaluated itemtotal correlation and refined it to nine items, achieving a Cronbach’s *α* of 0.89, indicating high internal consistency and reliability (21, 22). The scoring ranges from 0 to 10 points for each item, with a total score ranging from 0 to 90 for the nineitem scale. The score distribution is displayed in **Figure 2**. The average score for suicidal ideation across all subjects was 17.19 (*SD* = 7.01). To categorize the subjects into groups based on their level of suicidal ideation, we applied a threshold of mean plus standard deviation, a common practice in psychological research (23, 24). Consequently, 94 subjects were classified into the high suicidal ideation group, while the remaining 712 were categorized as low suicidal ideation.

For the Tree-Drawing Test, we selected 98 significant features that can be grouped into 12 classes according to the current psychological research (25–28). These features are shown in **Table 1**, they were manually labeled by three graduate students specializing in psychometrics. If a certain feature was present in an image, it was marked as “1”; if absent, it was marked as “0”. The labeling process was conducted independently by each annotator, resulting in an initial agreement rate of 94%. Any disagreements were resolved through discussion among annotators to reach consensus final labels. Notably, the data collection was conducted in accordance with the Declaration of Helsinki, and approved by the Institutional Review Board of Beijing Normal University (protocol code: 202112300092, and date of approval: December 30, 2021).

**Table 1:** Classes and details of tree-drawing features.

Class	Features
overall	huge tree; small tree; tree above; tree below; tree on the left; tree on the right; tilted trunk or crown; cut at the edge of the tree and paper; cut above; cut on the left; cut on the right; cut below
line	thick lines; light lines; obvious differences in strength; sloppy lines; mainly short lines; mainly long lines; mainly trembling wavy lines
special mark	snakelike; symmetrical
special tree type	dead tree; simplified tree; pine tree; theme loss; multiple trees
canopy	closed canopy; open canopy; zoned canopy; large canopy; small canopy; squashed canopy; full canopy; emphasized canopy lines; light canopy lines; multi-layered canopy lines; cloud-like canopy lines; ringed canopy line; quivering shaped canopy lines; circular canopy lines; black shadows in the canopy; chaos in the canopy; blank canopy; detailed depicted canopy; short wavy lines drawn in the canopy
branch	broken branches; crossing branches; vigorously growing branches; drooping branches; patchwork branches; single-line branches; parallel branches; open branch ends; sharp branch ends
leaf and flower	fruits or flowers in the canopy; leaves; fallen leaves
trunk	small trunk; long trunk; parallel trunk; wide at the top and narrow at the bottom; wide at the bottom of the trunk; scars on the trunk; black shadow on the trunk; depiction of bark; completely blank trunk; emphasis on trunk edge lines; light trunk edge lines; trunk with small twigs
junction	trunk sealed at the top; trunk open at the top; trunk forms a ‘M’ shape at the top; trunk sealed at the bottom; trunk transitionally sealed at the bottom (drawing whisker-like roots); trunk open at the bottom; trunk directly connects to the branches and the junction is hollow
root	drawn roots; fibrous roots; sharp roots; crossing roots; overly drawn roots; exposed roots
ground	drawn ground line; sloping ground line; wavy ground line; hilly ground line; emphasized ground; paper’s base as ground line (only if depicted flowers and plants); ground line through the trunk
attachment	drawn attachments; sun; clouds; flowers under the tree; grass under the tree; birds or bird’s nests; houses or people; words; wind and rain

### 2.2 Baselines

To identify the high suicidal ideation, any binary classifier of machine learning algorithms can be effectively applied to features, extracted by automatic tree features extraction model or manually labeled from images. In this study, we adopted some machine learning (ML) models, including Logistic Regression (LR) (29), Decision Tree (DT) (30), Support Vector Machine (SVM) (31) and Random Forest (RF) (32) to implement binary classification. Besides, the suicidal ideation detection on images can be realized by deep learning techniques directly. Convolutional Neural Networks (CNN) is a common framework in deep learning, and also widely used in image classification. We selected some classic CNN models, such as AlexNet (33), VGG16 (34), Inception (35) and ResNet (36) as our baselines. To make a comparison with state-of-the-art graph learning models, we also selected GAT (37), HAN (38) and Simple-HGN (S-HGN) (39) as baselines.

### 2.3 Proposed model

#### 2.3.1 Overview

**Figure 3:**
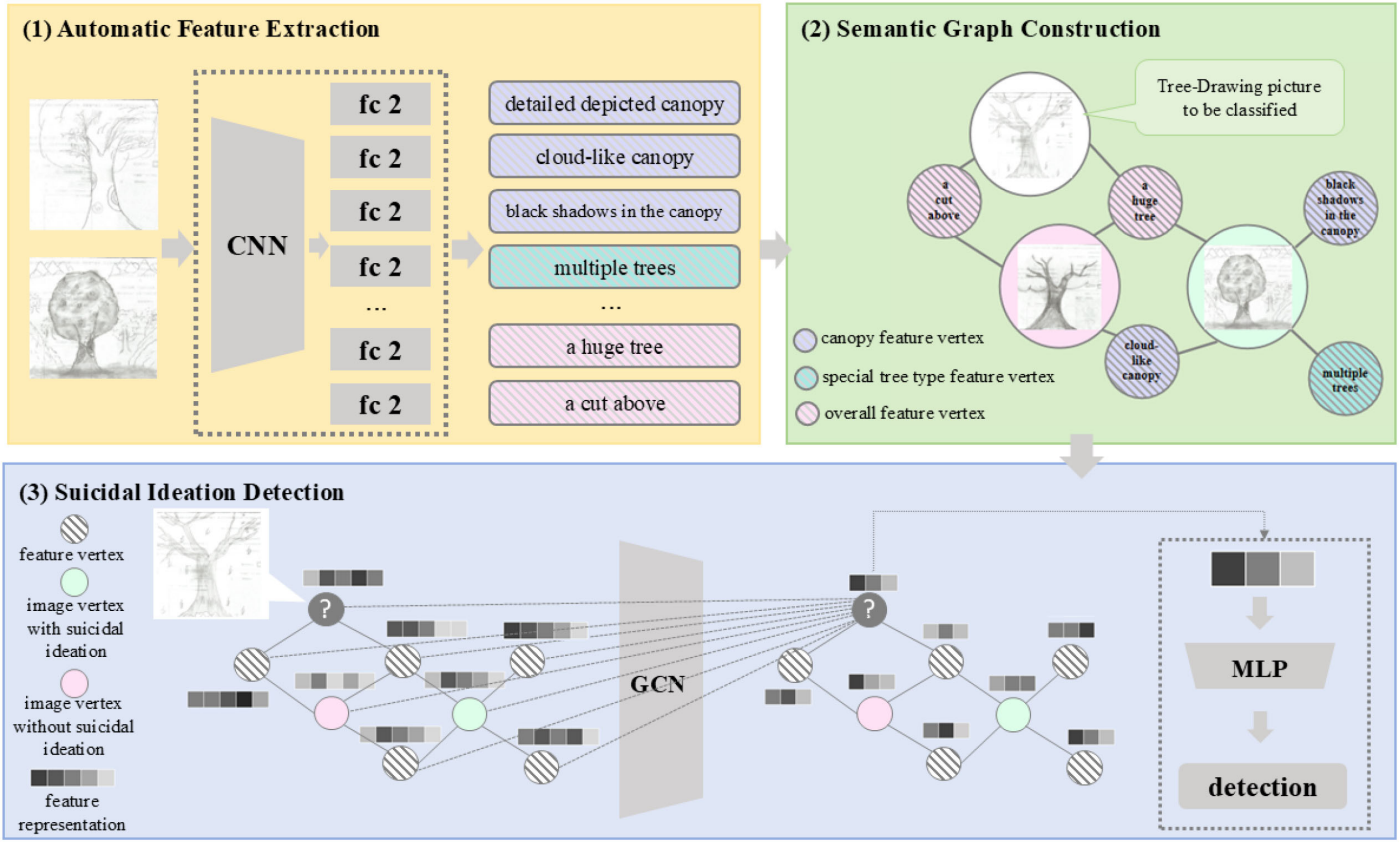
Overall framework of the proposed model, which consists of three modules: (1) automatic feature extraction, (2) semantic graph construction, and (3) suicidal ideation detection. Such a method formalizes individual suicidal ideation detection as a node classification task in graph. Based on graph learning, the images from Tree-Drawing Test can be better represented via combining psychological tree features and deep learning techniques, leading to a better performance on suicidal ideation detection.

In this study, we proposed a new model to achieve automatic suicidal ideation detection based on TreeDrawing Test, as illustrated in **Figure 3**. The model consists of three modules. The first one is automatic feature extraction, determining which tree features are contained in Tree-Drawing Test images. The second one is semantic graph construction, it aims at establishing a graph, where the weight of edge represents the correlation between psychological tree feature and individual image. The third one is suicidal ideation detection, which realizes the detection of individual suicidal ideation based on the Tree-Drawing Test and graph node classification. Such a method transforms traditional image classification task to graph node classification task. Based on Graph Convolutional Network (GCN), the images from Tree-Drawing Test can be better represented, leading to a better performance on suicidal ideation detection. For clearer presentation, **Table 2** displayed the main notations and descriptions.

**Table 2:** Notations and descriptions of the proposed model.

Symbols	Notations and descriptions
G	Image-feature semantic graph
V	Vertices set
$V_{F}$	Feature vertices set
$V_{I}$	Image vertices set
ℰ	Edge set
**A**	Adjacency matrix
**X**	Initial representation of all vertices
**H** ^(^ *^l^* ^)^	Feature representation at the *l*-th layer
**W** ^(^ *^l^* ^)^	Trainable weight matrix of the *l*-th layer
**y** *^j^*	The ground truth label for the *j*-th vertice
${\hat{y}}^{j}$	The prediction for the *j*-th vertice
*w_c_*	The weight of class *c*
$z_{i}^{j}$	The ground-truth of manual annotation whether the *j*-th image sample has the *i*-th feature
${\hat{z}}_{i}^{j}$ $w_{f}^{i}$	The probability that the model predicts the *j*-th image sample has the *i*-th feature The weight of minority class for the *i*-th feature

#### 2.3.2 Automatic tree features extraction

Based on the manually-labeled features, we fine-tuned a ResNet network to achieve automatic tree features extraction through multi-label classification of 98 image features. Specifically, we modified the output of the last fully-connected layer in ResNet34 (36) as 98 parallel linear layers. The model performed binary classification for each feature, and outputted a 98-dimensional vector that indicates whether the feature exists in the input image. It is trained by cross entropy loss, which is commonly used for classification tasks. For the *i*-th feature, the loss ${ℒ}_{feature}^{i}$ is shown in Equation 1. The total loss $L^{1}$ is the mean of the losses of each feature, as shown in Equation 2.


(1) $${ℒ}_{feature}^{i}=-\frac{1}{M}\sum_{j=1}^{M}({\text{z}}_{i}^{j}\text{log }({\hat{\text{z}}}_{i}^{j})+\left(1-{\text{z}}_{i}^{j}\right)\text{log }(1-{\hat{\text{z}}}_{i}^{j})).$$

(2) $${ℒ}^{1}=\frac{1}{N}\sum_{i=1}^{N}{ℒ}_{feature}^{i},i=1,2,\ldots ,N.$$

Here, ${\hat{z}}_{i}^{j}$ is the probability that the model predicts the *j*-th image sample has the *i*-th feature and $z_{i}^{j}$ represents the ground-truth of manual annotation whether the *j*-th image sample has the *i*-th feature. *M* is the number of training images, and *N* is 98 since there are 98 predefined tree features.

#### 2.3.3 Semantic graph construction

To link the above psychological tree features and images, a semantic graph is constructed and further used to explore intrinsic semantic information contained in images. Generally, a graph is composed of a finite number of vertices and edges between them, usually represented as G=(V,ℰ) , where G denotes the graph, V is the set of vertices in graph G , $v_{i}$ represents the *i*-th node and ℰ is the set of edges in graph G . In this model, we take both features and images as vertices to build the image-feature semantic graph, that is, $V=V_{F}\cup V_{I}$ , where $V_{F}$ and $V_{I}$ respectively represent the set of feature vertices and the set of image vertices. If the number of images and features are $\left|V_{F}\right|$ and $\left|V_{I}\right|$ respectively, then the total number of vertices is $\left|V\right|=\left|V_{F}\left|+\right|V_{I}\right|$ . Moreover, the edge set ℰ of semantic graph is generated according to the results of automatic tree features extraction. When the *i*-th image ( $v_{i}\in V_{I}$ ) has the *j*-th feature ( $v_{j}\in V_{F}$ ), there is an edge $e_{i,j}\in ℰ$ between image vertex $v_{i}$ and feature vertex $v_{j}$ in the graph G . In graph theory, the adjacency matrix is commonly used to represent the relationship between vertices. For semantic graph G=(V,ℰ) , the corresponding adjacency matrix is an |V| by |V| square matrix $A\in {\mathbb{R}}^{\left|V\right|\times \left|V\right|}$ with each element generated as Equation 3.


(3) $$A\left(i,j\right)=\{\begin{array}{ll} 1, & if\;e_{i,j}\in ℰ\\ 0, & \text{otherwise} \end{array},\text{where }i,j=1,2,\ldots ,\left|V\right|.$$

If there is an edge $e_{i,j}\in ℰ$ between vertex *v_i_* and vertex *v_j_* in graph G, then the element in the *i*-th row and the *j*-th column of adjacency matrix **A** is 1, otherwise it is 0. In this way, the semantic knowledge of Tree-Drawing Test images can be fully preserved in the semantic graph. Consequently, the semantic graph is composed of 904 nodes and 15379 edges, and the edge density is approximately 0.04, indicating that the semantic graph is sparse.

#### 2.3.4 Suicidal ideation detection based on tree-drawing test and graph learning

Since each image vertex has its label, where images of the individuals with low suicidal ideation belong to class 0 and the others belong to class 1, the suicidal ideation detection is transformed into a node classification task on the constructed semantic graph. The graph convolutional network (GCN) has a great expressive power to learn the node representations and has achieved a superior performance in node classification (40). Therefore, the graph convolutional network is adopted to achieve the final classification results by the following three steps:

##### 2.3.4.1 Initial representation

Fundamentally, a GCN takes a graph together with a set of feature vectors as input, where each node is associated with its own feature vector. In this model, the initial feature vector is obtained by node2vec (41), which learns low-dimensional embedding for nodes in image-feature semantic graph via applying random walks on semantic graph starting at a target node. Specifically, it first calculates the transition probabilities between nodes, then generate the walk sequence by biased random walk, and finally obtains the representation through the Skip-gram method (42). It enables structurally-similar vertices to have similar representations. Assume that the feature of each vertex is represented as a *k*-dimensional vector, finally we obtained the set of all vertices representations $X\in {\mathbb{R}}^{\left|V\right|\times k}$ .

##### 2.3.4.2 Graph convolutional network for node representation

To integrate the information of psychological tree features (i.e. the feature vertices) into the image representations (i.e. the embedding of image vertices), we leveraged Graph Convolutional Networks (GCN) (43), which can learn both topology structure of the graph and semantic features of vertices. GCN implements the node representation process as shown in Equation 4.


(4) $$H=f_{\theta }\left(X,A\right).$$

**Figure 4:**
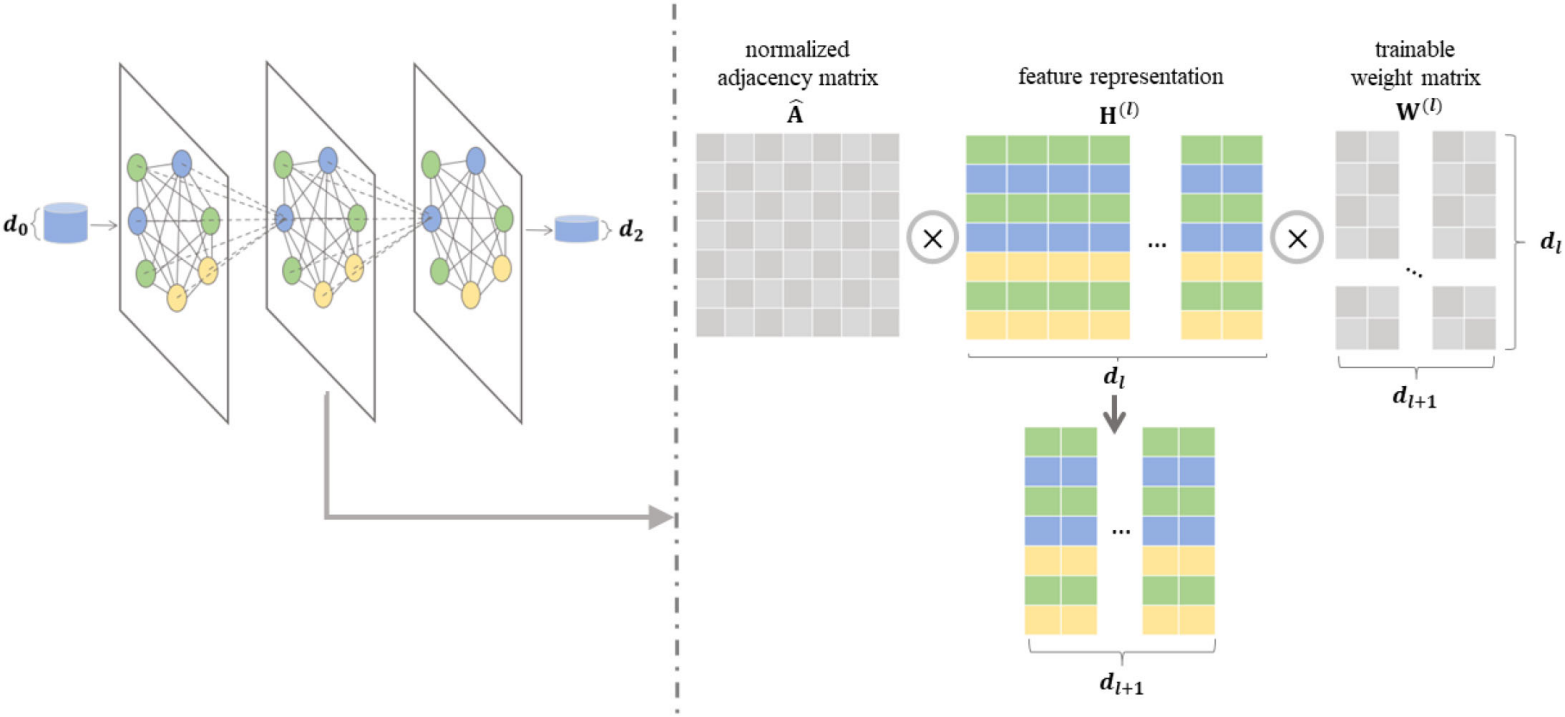
The process and details of graph convolutional network (GCN). Left: GCN can update the embeddings of vertices in the graph based on the known adjacency matrix and vertex embeddings. Right: The *l*-th layer of GCN output **H** ^(^ *^l^* ^+1)^ by calculating product of ${\text{H}}^{\left(l\right)},\hat{\text{A}}$ and **W** ^(^ *^l^* ^)^.


Here, **X** is the initial vertex embeddings, *θ* is the learnable parameter in GCN model, **A** is the adjacency matrix of image-feature semantic graph, and **H** ^(^ *^L^* ^)^is the representation of all unlabeled image vertices. The key of GCN model is to obtain a good node representation. To achieve this, GCN can update the embedding of vertex by aggregating the information from its corresponding neighbors via a multi-layer structure, as shown in **Figure 4**. If GCN model has *L* layers, every GCN layer updates the node features according to Equation 4.

(5) $$H^{\left(l+1\right)}=\sigma \left(\hat{A}H^{\left(l\right)}W^{\left(l\right)}\right),\text{where }l=0,1,\ldots ,L-1,$$

where $\hat{A}\in {\mathbb{R}}^{\left|V\right|\times \left|V\right|}$ is the normalized adjacency matrix calculated by $\hat{A}=D^{-\frac{1}{2}}AD^{-\frac{1}{2}}$ , **D** represent graph degree matrix, which is a diagonal matrix with each diagonal element as $D\left(i,i\right)=\sum_{i=1}^{\text{|V|}}A\left(i,j\right)$ . And $W^{\left(l\right)}\in {\mathbb{R}}^{d_{l}\times d_{l+1}}$ is a trainable weight matrix of the *l*-th layer and *σ* represents an activation function. $H^{\left(l\right)}\in {\mathbb{R}}^{\left|V\right|\times d_{l}}$ is the feature representation at the *l*-th layer. Notably, the initial state, $H^{\left(0\right)}$ , is set as **X**, i.e., $H^{\left(0\right)}=X$ .

##### 2.3.4.3 Node classification

The output of the *L*-th GCN layer is **H** ^(^ *^L^* ^)^, and node classification is realized by applying a fully-connected layer with softmax activation function on it as formulated by Equation 6.


(6) $$y=softmax\left(WH^{\left(L\right)}+b\right),$$

where $W\in {\mathbb{R}}^{2\times d_{L}}$ , $b\in {\mathbb{R}}^{2\times 1}$ , $y\in {\mathbb{R}}^{\left|V\right|\times 1}$ . The parameters of the GCN are trained by the commonly-used cross entropy loss as shown in Equation 7.


(7) $${ℒ}^{2}\left(\hat{y},y\right)=-\frac{1}{T}\sum_{j=1}^{T}(y^{j}\text{log }({\hat{y}}^{j})+\left(1-y^{j}\right)\text{log }(1-{\hat{y}}^{j})),$$

where $\hat{y}$ and **y** are the predicted values and ground truth labels of image vertices, that is the prediction and real labels of Tree-Drawing Test image about suicidal ideation, respectively. *T* is the number of training image vertices with labels in the semantic graph. ${\hat{y}}^{j}$ indicates the prediction for the *j*-th vertice. The whole GCN model is optimized through back propagation.

#### 2.3.5 Cost-sensitive strategy for class-imbalanced issue

It is worth noting that the number of individuals with high suicidal ideation is usually much smaller than that of individuals with low suicidal ideation, so there is an class-imbalance issue for suicidal ideation detection. Similarly, automatic tree feature extraction also suffer from class-imbalance issue. To address issue of the class-imbalanced distribution during both automatic feature extraction and suicidal ideation detection, cost-sensitive strategy is employed by leveraging weighted cross entropy loss, which is shown in Equation 8. By giving minority class with a larger weight, cost-sensitive strategy would penalize more if incorrect prediction is achieved for these minority class. As a result, the prediction accuracy for these minority class is improved. For the binary classification process of suicidal ideation detection, by using cost-sensitive strategy, the loss function of GCN model in Equation 7 is revised as follows:


(8) $${ℒ}_{imbalance}^{2}\left(\hat{\text{y}},\text{y}\right)=-\frac{1}{T}\sum_{j=1}^{T}(w_{c}{\text{y}}^{j}\text{log }({\hat{\text{y}}}^{j})+\left(1-{\text{y}}^{j}\right)\text{log }(1-{\hat{\text{y}}}^{j})).$$

Here, *w_c_* is the weight of class *c*, where the *j*-th sample belongs to, i.e., *c* = **y** *^j^*. When the *j*-th image vertice represents sample with high suicidal ideation (class 1 and **y** *^j^* = 1), a larger weight *w*_1_ is assigned to the minority class sample. Through assigning a larger weight *w_c_* for each sample in minority classes, the penalty on minority class in the loss function can be adjusted to alleviate the class-imbalanced issues and further detect more effective individuals with high suicidal ideation as well as tree features.

Similarly, in the multi-label classification process of automatic tree feature extraction, the cost-sensitive strategy is employed to handle the class-imbalanced tree features distribution, the loss function of automatic tree features extraction model in Equation 1 for the *i*-th feature is revised as follows:


(9) $${ℒ}_{feature,imbalance}^{i}=-\frac{1}{M}\sum_{j=1}^{M}M(w_{f}^{i}z_{i}^{j}\text{log }({\hat{z}}_{i}^{j})+\left(1-z_{i}^{j}\right)\text{log }(1-{\hat{z}}_{i}^{j})).$$

Here, $w_{f}^{i}$ is the weight of minority class for the *i*-th feature. For the *i*-th feature classification, if images with the *i*-th feature belongs to the minority class, a larger weight $w_{f}^{i}$ is assigned to images with label $z_{i}^{j}=1$ . If images without *i*-th feature belongs to the minority class, a larger weight weight $w_{f}^{i}$ is assigned to images without *i*-th feature, i.e., images with label $z_{i}^{j}=0$ .

### 2.4 Experimental settings

Lots of experiments are conducted to validate the effectiveness of the proposed method. These experiments are designed to address the following research questions:

- *RQ-1*: Does the proposed graph learning method outperform other baselines?
- *RQ-2*: Can automatic tree features extraction replace manual annotation for the suicidal ideation detection task?
- *RQ-3*: Does each class of tree features contribute to the individual suicidal ideation detection?
- *RQ-4*: How do different hyperparameters (i.e. cost-sensitive weight for suicidal ideation detection, cost-sensitive weight for feature extraction, and the number of layers in GCN) affect the performance of suicidal ideation detection?
- *RQ-5*: Does the graph learning method perform stable on different sizes of training set?

When comparing the proposed method with the baselines, features are firstly extracted by automatic feature extraction module for each image. Then, an image can be represented as a 98-dimensional vector, where each dimension represents the presence or absence of the corresponding feature. For ML models, classification results are obtained by applying ML models on the extracted 98-dimensional vector. For the graph learning model represented by GCN, the extracted tree features are used to build the image-feature semantic graph, and then the model is applied for suicidal ideation detection task. In graph learning models, such as HAN and Simple-HGN, the image-feature semantic graph is represented as a heterogeneous graph, where image nodes and feature nodes are treated as two distinct types of nodes. The two types of edges are “image-has-feature” and “feature-exists in-image.” For CNN models, suicidal ideation detection can be seen as a simple image classification task with original image as input.

Additionally, **Table 3** shows the hyperparameters in the proposed method. Specifically, we used a multi-label learning model to implement automatic tree-drawing features extraction. By using the manuallyannotated features as labels, we trained the automatic tree features extraction model to obtain feature predictions for each image. During the training, the learning rate is set as 0.01, and the weight decay is set as 0.000001. For the class-imbalance issue in automatic tree feature extraction, the cost-sensitive weighted hyperparameter ( $w_{f}^{i}$ in Equation 9) is set to 4.0. Also, a two-layer GCN (*L* = 2) is employed as graph learning module, and the ReLU function is used as the activation function in Equation 5. To train GCN model, the learning rate is set as 0.01 and the weight decay is 0.001. For the class-imbalance issue in suicidal ideation detection, the cost-sensitive weighted hyperparameter (*w_c_* in Equation 8) is set to 7.0, These hyperparameters are determined by sensitivity analysis as shown in section 3.4. Besides, after the semantic graph construction, the graph contains 904 vertices(|*V*| = 904), comprising 806 image vertices and 98 feature vertices. For performance evaluation, 80% of image vertices are used as the training set and the rest 20% of image vertices are utilized as the testing set. The performance on the testing set is reported.

**Table 3:** Hyperparameter settings.

Hyperparameter	Description	Value
*L*	The number of GCN layers.	2
activation function	A mathematical function used in neural networks to introduce non-linearity, allowing the network to learn complex patterns.	ReLU
learning rate	A hyperparameter that controls the step size of parameter updates during model training.	0.01
weight decay	A technique used to prevent overfitting by adding a penalty term to the loss function.	0.001
*w_c_*	The weight of class c.	7.0
$w_{f}^{i}$	The weight of minority class for the i-th feature.	4.0

### 2.5 Metrics

Regarding the task of suicidal ideation detection, it is more important to minimize missed cases with high suicidal ideation. Since suicidal ideation detection suffers from serious classimbalanced issue, the individuals who actually have high suicidal ideation (class 1) but are predicted as low suicidal ideation (class 0) are our main concern. Therefore, the following metrics, widely used in imbalanced classification, are employed to evaluate performance: precision of class 0 (precision_0_), recall of class 1 (recall_1_), macro average of F1 score (macro-F1), G-mean and false positive rate (FPR). They are defined in Equations 10–14 as follows (44):


(10) $${\text{precision}}_{0}=\frac{\text{TN}}{\text{TN}+\text{FN}},\;{\text{recall}}_{0}=\frac{\text{TN}}{\text{TN}+\text{FP}},$$

(11) $${\text{precision}}_{1}=\frac{\text{TP}}{\text{TP}+\text{FP}},\;{\text{recall}}_{1}=\frac{\text{TP}}{\text{TP}+\text{FN}},$$

(12) $$\begin{array}{c} \text{F}1_{0}=2\times \frac{\text{precisio}{\text{n}}_{0}\times \text{recal}{\text{l}}_{0}}{\text{precisio}{\text{n}}_{0}+\text{recal}{\text{l}}_{0}},\\ \text{ F}1_{1}=2\times \frac{\text{precisio}{\text{n}}_{1}\times \text{recal}{\text{l}}_{1}}{\text{precisio}{\text{n}}_{1}+\text{recal}{\text{l}}_{1}},\\ \text{ macro}-\text{F}1=\frac{1}{2}\times (\text{F}1_{0}+\text{F}1_{1}), \end{array}$$

(13) $$\text{G}-\text{mean}=\sqrt{\frac{\text{recal}{\text{l}}_{1}\times \text{TN}}{\text{TN}+\text{FP}}},$$

(14) $$\text{FPR}=\frac{\text{FP}}{\text{FP}+\text{TN}},$$

where TP, TN, FP, and FN denote true positive, true negative, false positive, and false negative respectively. F1_0_ and F1_1_ represent F1 scores of class 0 and class 1 respectively. Macro-F1 can evaluate the model’s performance by treat all classes equally. G-mean is a comprehensive indicator of the recall of class 0 and class 1. For the above metrics, a higher value indicates better performance. The False Positive Rate (FPR) measures the model’s tendency to incorrectly predict negative samples as positive. A lower FPR indicates better performance in identifying negative samples.

## 3 Results

### 3.1 The proposed GCN method achieves better performance than baselines (RQ-1)

**Figure 5:**
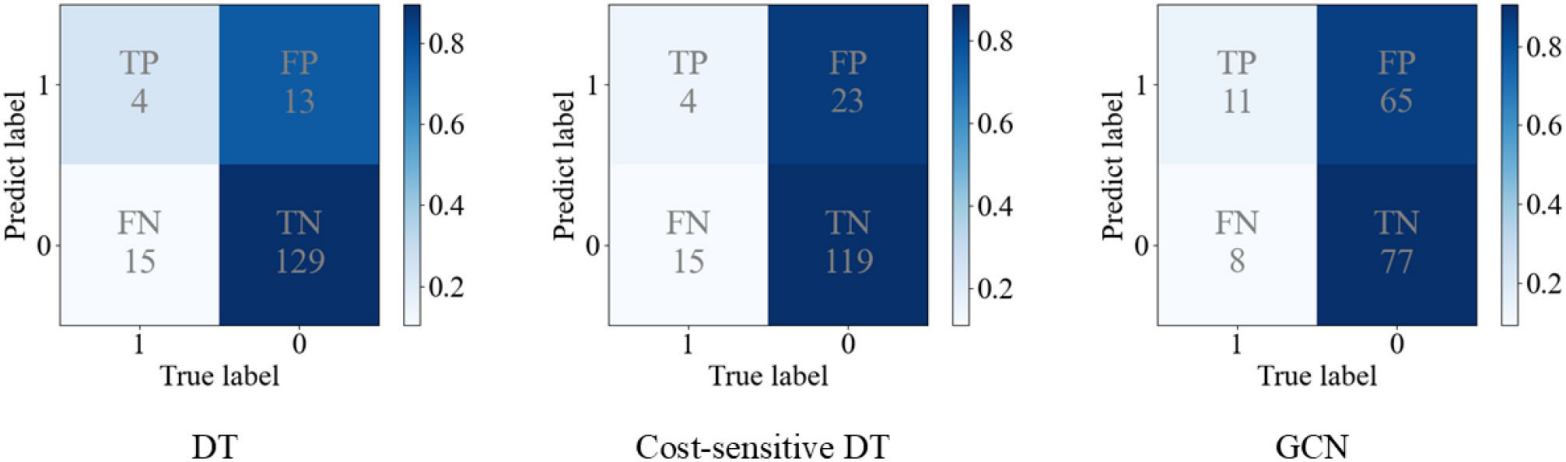
The confusion matrices of decision tree (DT), cost-sensitive DT and Graph Convolutional Network (GCN).

The performance of all methods are reported in **Table 4** (left), with the cost-sensitive strategy consistently applied to both CNN models and graph learning models. From the results, both ML models and graph learning models have better result on macro-F1, demonstrating that these models perform well in predicting both high suicidal ideation class and low suicidal ideation class. Different from CNN models, which use convolution operations to extract the features of images, ML models and graph learning models were conducted based on the 98 tree-drawing features extracted from the images (see Section 2.3.2). Since these tree-drawing features are meaningful psychologically, they are relevant to individual suicidal ideation. They are well-suited for the task of suicidal ideation detection, thereby improving the overall performance. In order to explain the results more intuitively, **Figure 5** shows the confusion matrices of decision tree (DT), cost-sensitive DT (45) and GCN. It can be noticed that the TP of GCN is higher than that of DT, thereby the recall_1_ is higher. Since recall_1_ is a very important metric in our task, which measures the model’s ability to recognize the individuals with high suicidal ideation, the ML methods are not competitive for this task. In this case, lower FPR is due to a small number of high suicidal ideation being identified. The highest G-mean also demonstrates this point. Conceivably, compared with CNN and ML models, different tree-drawing image nodes are connected through tree-drawing feature nodes in semantic graph, similarities and differences among different samples during training can be better captured by graph.

**Table 4:** Performance comparison results.

model	precision_0_	recall_1_	macro-F1	G-mean	FPR	model*	precision_0_	recall_1_	macro-F1	G-mean	FPR
LR	89.36%	21.05%	54.78%	43.22%	11.27%	LR*	89.93%	26.32%	56.68%	48.13%	11.97%
DT	89.58%	21.05%	**56.22%**	43.73%	9.15%	DT*	89.15%	26.32%	52.24%	46.17%	19.01%
SVM	88.96%	10.53%	53.98%	31.87%	**3.52%**	SVM*	90.13%	21.05%	60.88%	45.07%	3.52%
RF	88.96%	10.53%	53.98%	31.87%	**3.52%**	RF*	89.03%	10.53%	54.46%	31.98%	2.82%
AlexNet	88.17%	42.11%	44.09%	49.31%	42.25%	–	–	–	–	–	–
VGG16	86.89%	**57.89%**	35.35%	46.49%	62.68%	–	–	–	–	–	–
Inception	86.44%	**57.89%**	34.46%	45.60%	64.08%	–	–	–	–	–	–
ResNet	85.29%	47.37%	35.65%	43.99%	59.15%	–	–	–	–	–	–
GAT	89.74%	**57.89%**	42.60%	53.42%	50.70%	GAT*	91.09%	52.63%	50.52%	58.39%	35.21%
HAN	87.95%	47.37%	41.72%	49.35%	48.59%	HAN*	90.72%	52.63%	48.87%	57.11%	38.03%
S-HGN	88.76%	47.37%	44.09%	51.34%	44.37%	S-HGN*	90.00%	42.11%	50.71%	54.18%	30.28%
GCN	**90.59%**	**57.89%**	45.50%	**56.03%**	45.77%	GCN*	92.68%	52.63%	60.56%	65.00%	19.72%

Bold indicates the best result among all methods. *represents the situations where the tree features are manually-annotated.

-represents the situations where the model implementation does not rely on manually-annotated tree features.

As for the comparison with other graph learning models, GCN outperforms in terms of all metrics. We hypothesize that, due to the limited number of node types and edge types in the image-feature semantic graph, heterogeneous graph learning models are unable to fully demonstrate their advantages. Compared to GAT, which assigns adaptive edge weights through attention mechanisms, GCN still demonstrates superior performance. This discrepancy may suggest that maintaining equal edge weights is more suitable for the current task. Additionally, this could indicate that the current dataset size might not be sufficient for graph learning to learn the feature importance. Further exploration is warranted with a larger dataset in the future research.

### 3.2 Automatic extraction of tree-drawing features has room for improvement (RQ-2)

As mentioned in Section 2.3, we used a multi-label learning model to implement automatic tree-drawing features extraction. On the basis, we compared the models using automatically-extracted tree features with that using manually-annotated tree features for individual suicidal ideation detection. The results are reported in **Table 4** (left vs. right). For a specific model, the suicidal ideation detection performance with manually-annotated tree features can be considered as idealized result. If the performance obtained using automatically-extracted tree features is closer to the idealized result, it means automatic feature extraction is more effective. From the results, it is apparent that individual suicidal ideation detection depends on accurate tree features for both machine learning models and graph learning models. In our method, the tree-drawing features are represented as vertices, which only have connections with image vertices in the image-feature semantic graph. The representation of a tree-drawing image would heavily be affected by the quality of features based on the information propagation mechanism of GCN, leading to significant improvement in terms of macro-F1 and G-mean metrics with manually-annotated feature for GCN. Thus, there is still significant room for improvement in automatic feature extraction in the future. Besides, it can be seen that GCN model outperforms ML models in suicidal ideation detection even under the setting of using manually-annotated tree features, demonstrating the effectiveness of the proposed method again. In an ideal scenario, the performance of GCN still surpasses that of the ML models, CNN models, and other graph learning models. And, the ideal GCN model performs well in both macro-F1 (60.56%) and G-mean (65.00%), and also demonstrates good performance in recall_1_ (52.63%) and FPR (19.72%). This indicates that the GCN model has the highest potential for suicidal ideation detection based on Tree-Drawing Test.

### 3.3 All classes of tree-drawing test features can contribute to suicidal ideation detection verified by ablation study (RQ-3)

As above-mentioned in **Table 1**, there is a total of 12 features classes. In order to prove the effectiveness of the features we selected, we conducted an ablation experiment. Based on the manually-annotated tree features, we removed each class of features respectively, and further conducted a GCN-based suicidal ideation detection experiment with the remaining features. The results are shown in **Table 5**. The “removed feature” column represents the removed feature class, and the first “none” row means that all features are retained. The results indicate that the performance of the model after removing a certain class of features is slightly inferior to that without removing any class of features, which can be explained to a certain extent that all 12 classes of features are effective for individual suicidal ideation detection. Moreover, it is worth noting that when we removed the feature class “leaf and flower”, the performance had the most significant decline. In contrast, the performance reduction caused by removing feature class “root” or “attachment” is not obvious. We can initially conclude that for the task of suicidal ideation detection, features in the class “leaves and flowers”, such as “leaves” and “fruits”, play a more important role in detecting suicidal ideation than other features. In existing tree-drawing test studies, researchers have identified numerous characteristics related to psychological states, such as the shape of the tree crown, the inclination of the trunk, and the overall size of the tree (16, 46). The importance of “leaves and fruits” validated by our study provides a new perspective to this field. In terms of metric recall_1_, the value increased when the feature classes “special tree type”, “canopy”, “leaf and flower” or “ground” were removed, which may suggest that these features usually appear in the images of low suicidal ideation. In total, all the 12 classes of features have a certain effect on detecting individual suicidal ideation.

**Table 5:** Feature ablation experiment.

removed feature	precision_0_	recall_1_	macro-F1	G-mean	FPR
**none**	**92.68%**	52.63%	**60.56%**	**65.00%**	**19.72%**
overall	92.04%	52.63%	55.71%	62.09%	26.76%
line	91.89%	52.63%	54.81%	61.49%	28.17%
special mark	92.11%	52.63%	56.17%	62.38%	26.06%
special tree type	92.79%	**57.89%**	56.65%	64.80%	27.46%
canopy	92.45%	**57.89%**	54.38%	63.21%	30.99%
branch	91.67%	52.63%	53.49%	60.58%	30.28%
leaf and flower	90.91%	**57.89%**	46.74%	57.11%	43.66%
trunk	91.82%	52.63%	54.37%	61.18%	28.87%
junction	91.89%	52.63%	54.81%	61.49%	28.17%
root	92.62%	52.63%	60.04%	64.72%	20.42%
ground	92.73%	**57.89%**	56.19%	64.49%	28.17%
attachment	92.62%	52.63%	60.04%	64.72%	20.42%

Bold indicates the best result among all methods.

### 3.4 Sensitivity analysis helps determining the best hyperparameters (RQ-4)

#### 3.4.1 Cost-sensitive weight for suicidal ideation detection

**Figure 6:**
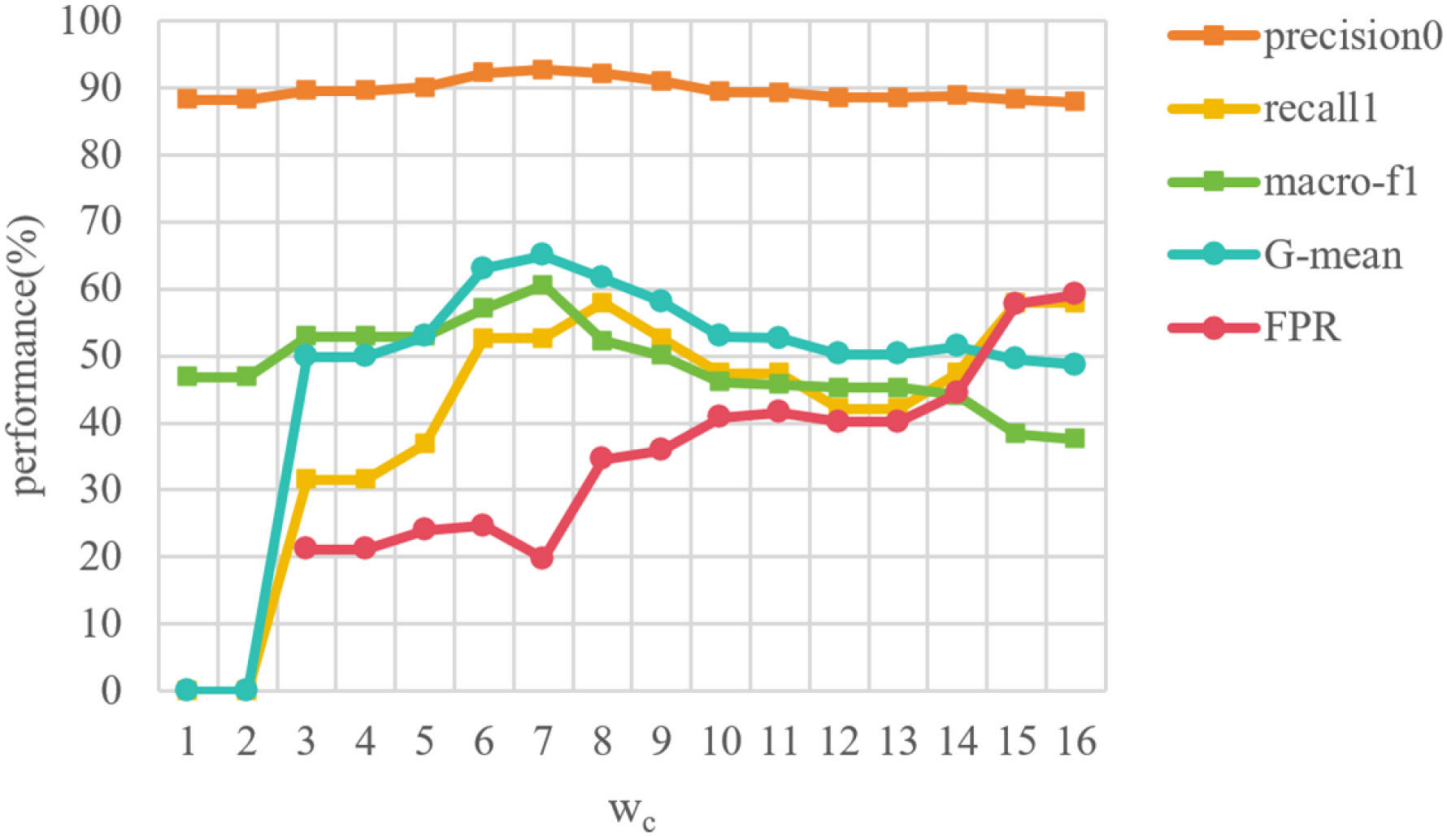
Sensitivity test of cost-sensitive weight for individual suicidal ideation detection.

In reality, the number of individuals with low suicidal ideation is much larger than that with high suicidal ideation, resulting in a serious class-imbalance issue between class 0 (low suicidal ideation) and class 1 (high suicidal ideation) in the dataset. A cost-sensitive strategy with weighting factor *w_c_* is introduced in Equation 8 to solve this problem. We tested the weight values in the range between 1 and 16 in steps of 1, since the ratio of samples with low suicidal ideation to those with high suicidal ideation is approximately 7.57:1. The performance with the change of weighting factor *w_c_* is shown in **Figure 6**. When the *w_c_* is 1 or 2, the values of G-mean and recall_1_ are zero, which means that the model has difficulties in detecting the samples with high suicidal ideation, and the model gives wrong predictions to all samples with high suicidal ideation. In general, the overall performance increases firstly and then decreases. The model has a more stable performance when the *w_c_* is between 6 and 9. Besides, it seems that the four metrics achieve the best results when *w_c_* is 7. It suggests that the proposed method requires selecting *w_c_* based on data distribution in practical applications.

#### 3.4.2 Cost-sensitive weight for feature extraction

**Figure 7:**
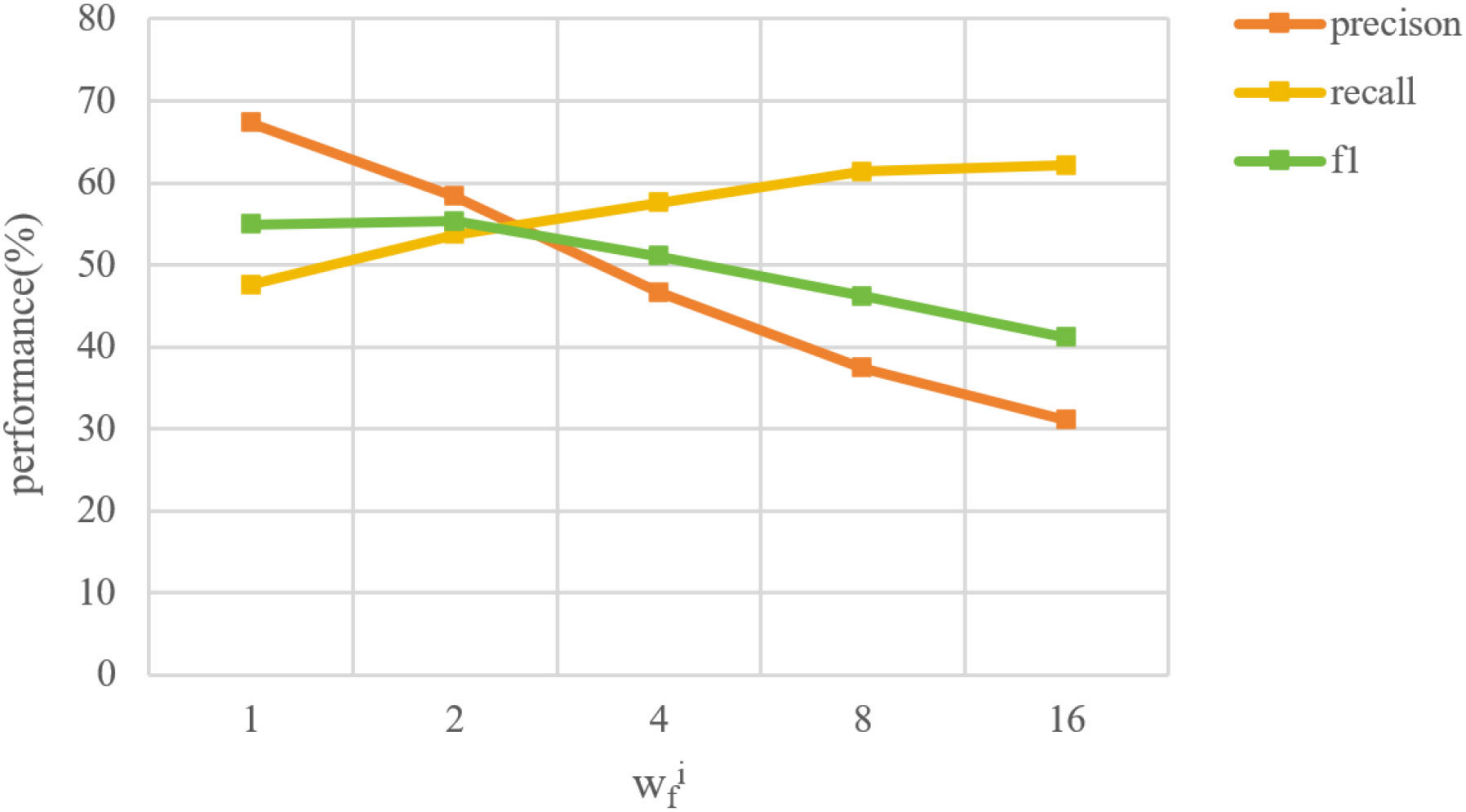
Sensitivity test of cost-sensitive weight for automatic features extraction.

For each feature, there is also a class-imbalance issue. For example, closed tree canopy is more common in tree images, so there are more images with the “closed canopy” feature than that without it in our dataset. And only a very small number of images contain houses or people, so the number of images without “houses or people” feature is much larger than that with it. Therefore, we introduced the weighting factor (named $w_{f}^{i}$ ) in Equation 9 during the automatic features extraction. For each feature, we gave a larger $w_{f}^{i}$ to the feature class with fewer samples when performing the multi-classification task. Following the same range of *w_c_* in **Figure 6**, we tried 5 different $w_{f}^{i}$ values for each feature class and conducted the automatic tree features extraction experiment, and the results are shown in **Figure 7**. The specific approach is using the manually-annotated features as labels and applying automatic tree features extraction model. During the training, $w_{f}^{i}$ is applied to the class with fewer samples for each feature. Overall, the feature extraction model performs the best when $w_{f}^{i}$ is 4.0.

After we obtained automatic feature extraction results based on the models with five different $w_{f}^{i}$ , the image-feature semantic graph is constructed and the suicidal ideation task is conducted respectively. The results are shown in **Table 6**. It can be noticed that GCN and DT perform better when $w_{f}^{i}$ is 4, and the best $w_{f}^{i}$ for LR, SVM and RF is 2. From the results, we can find that the performance with $w_{f}^{i}$ as 1 is worse than that of 2, 4 and 8, such result demonstrates that the cost-sensitive strategy does improve the performance of individual suicidal ideation detection. Meanwhile, there are opposite effects when the $w_{f}^{i}$ is too large.

**Table 6:** Suicidal ideation detection based on automatic feature coding with different *w_f_*.

Model	*w_f_*	Precision_0_	Recall_1_	Macro-F1	G-mean	FPR
GCN	1	89.36%	47.37%	46.06%	52.93%	40.85%
	2	90.32%	52.63%	47.24%	55.80%	40.85%
	4	90.59%	57.89%	45.50%	56.03%	45.77%
	8	90.00%	52.63%	46.02%	54.79%	42.96%
	16	89.58%	47.37%	46.85%	53.56%	39.44%
LR	1	89.29%	21.05%	54.33%	43.05%	11.97%
	2	88.71%	26.32%	50.28%	45.15%	22.54%
	4	89.36%	21.05%	54.78%	43.22%	11.27%
	8	88.54%	5.26%	50.84%	22.70%	2.11%
	16	89.24%	10.53%	56.09%	32.33%	0.70%
DT	1	88.74%	10.53%	52.63%	31.52%	5.63%
	2	88.81%	15.79%	52.67%	37.58%	10.56%
	4	89.58%	21.05%	56.22%	43.73%	9.15%
	8	88.98%	26.32%	51.44%	45.76%	20.42%
	16	84.47%	15.79%	39.41%	31.10%	38.73%
SVM	1	88.61%	5.26%	51.21%	22.78%	1.41%
	2	88.96%	10.53%	53.98%	31.87%	3.52%
	4	88.67%	5.26%	51.61%	22.86%	0.70%
	8	88.08%	5.26%	48.84%	22.20%	6.34%
	16	88.75%	5.26%	52.02%	22.94%	0%
RF	1	88.96%	10.53%	53.98%	31.87%	3.52%
	2	88.96%	10.53%	53.98%	31.87%	3.52%
	4	88.54%	5.26%	50.84%	22.70%	2.11%
	8	88.82%	10.53%	53.06%	31.63%	4.93%
	16	87.86%	10.53%	48.62%	30.20%	13.38%

#### 3.4.3 The number of GCN layers

Since the number of layers in GCN also has great influence in the performance of our suicidal ideation detection model, we experimented on GCN with 1, 2, 3, and 4 layers. The results are shown in **Table 7**. It shows that the best performance is achieved when the number of layers is 2. Such a result is also consistent with previous researches that stacking more layers in GCN will lead to worse performance, due to vanishing gradients and over-smoothing (47).

**Table 7:** The performance of the GCN with different number of layers.

Layer number	Precision_0_	Recall_1_	Macro-F1	G-mean	FPR
1	92.66%	57.89%	55.73%	64.17%	28.87%
2	92.68%	52.63%	60.56%	65.00%	19.72%
3	92.11%	52.63%	56.17%	62.38%	26.06%
4	91.67%	52.63%	53.49%	60.58%	30.28%

### 3.5 The Proposed GCN model exhibits stability as training data changes (RQ-5)

#### 3.5.1 Stability of the model with different training sizes

**Figure 8:**
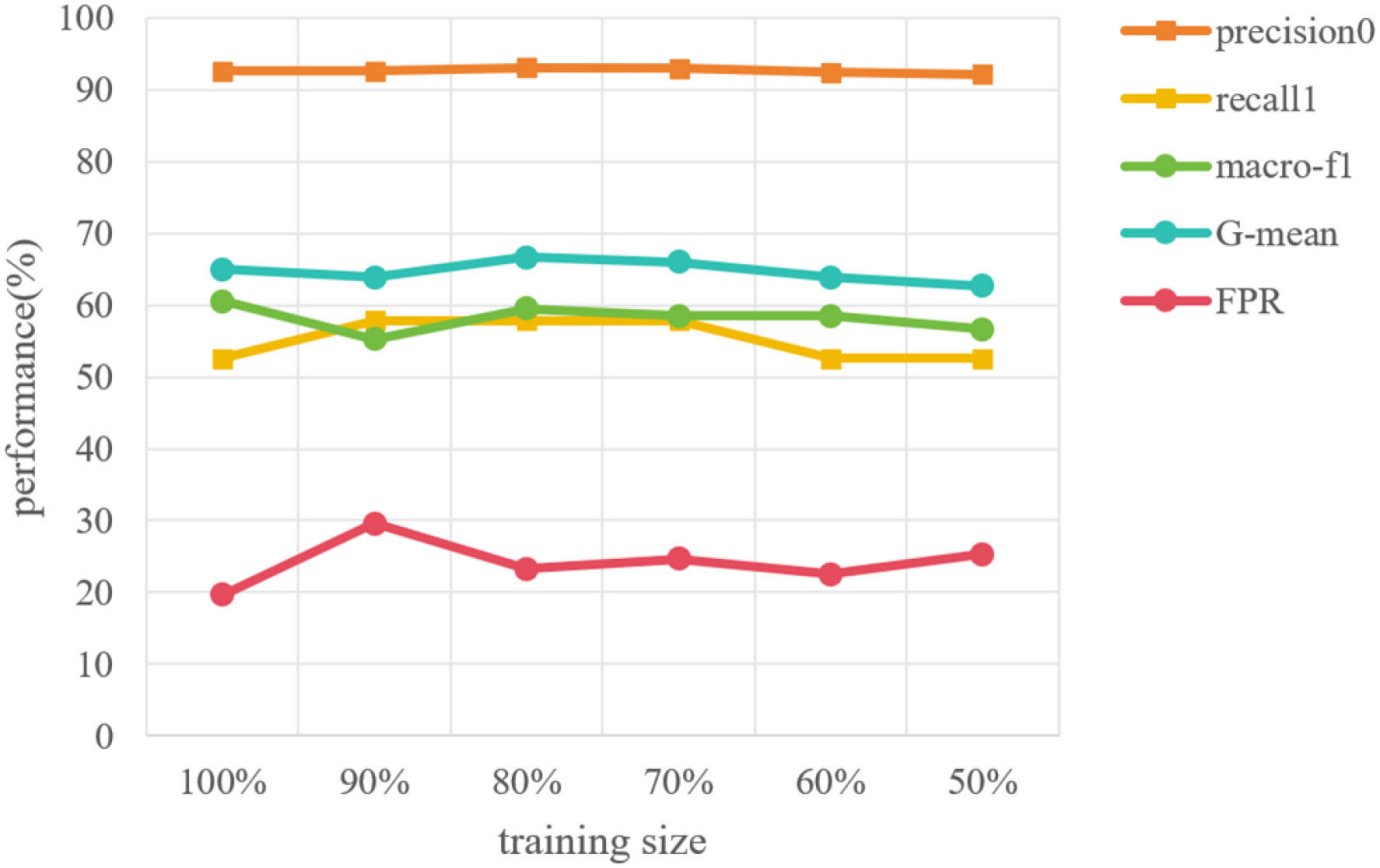
The performance of the model on training sets with different sample sizes.

In the suicidal ideation task, the image vertices on the graph are divided into 645 training vertices and 161 testing vertices, and GCN in graph learning module is trained with training data via back-propagation. Therefore, in order to show the stability of our proposed method with different training sizes, we conducted the experiments with different size of training data. Keeping the 161 testing vertices unchanged, 580 (90%), 516 (80%), 451 (70%), 387 (60%), 322 (50%) training vertices are randomly selected from 645 training vertices and used to construct the image-feature semantic graph respectively. Then the GCN model is trained on these graphs separately. The results are shown in **Figure 8**. It is easy to find that the experiment results do not fluctuate as the number of training vertices changes, which effectively proves the stability of the model.

#### 3.5.2 Stability of the model on different edge sizes

**Figure 9:**
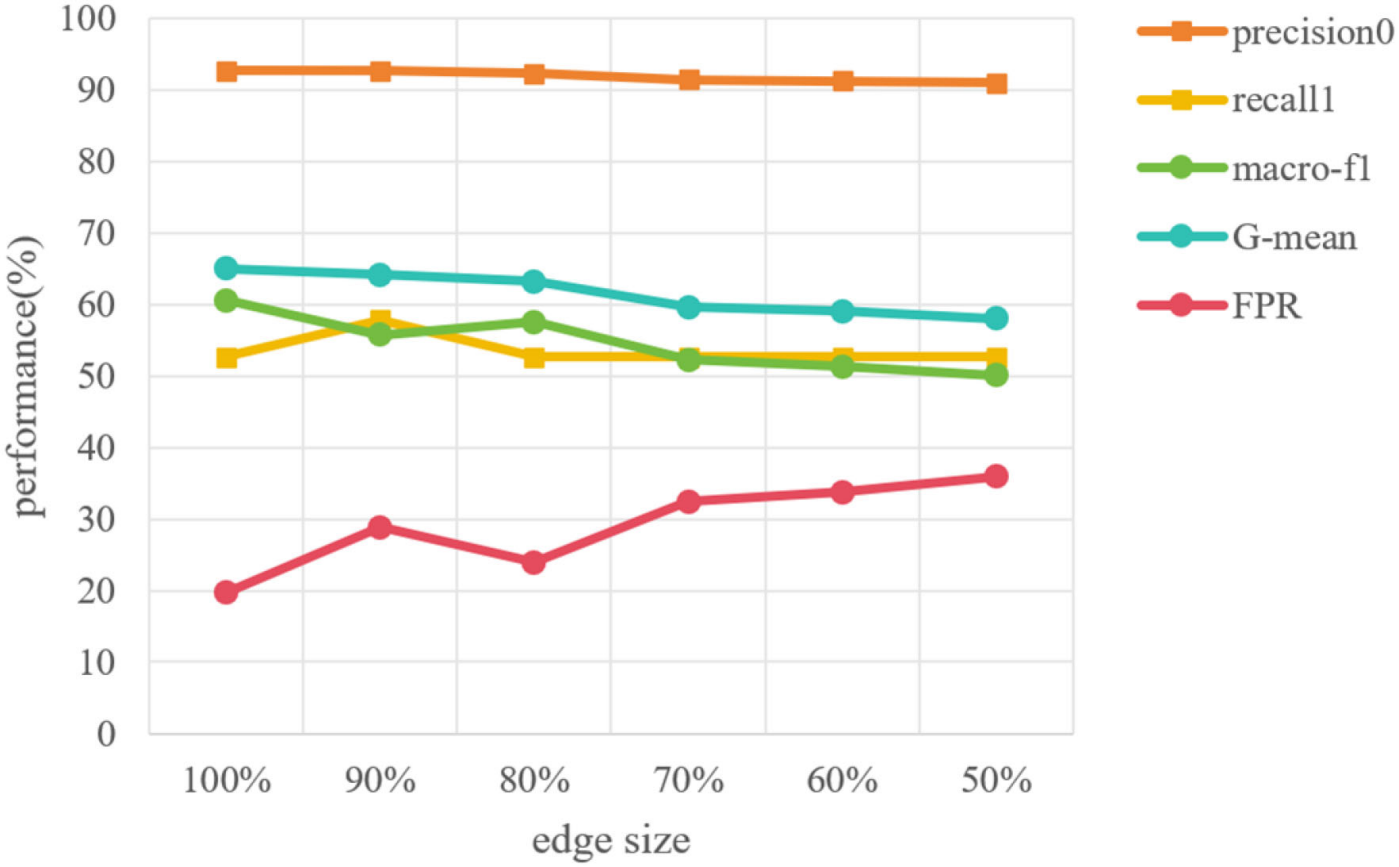
The performance of the model with different numbers of missing edges.

Considering the situation where insufficient individual expression or inaccurate automatic feature extraction leads to missing features, the semantic graph may miss some edges, i.e., the connections between tree-drawing image and its features. Thus, it is also necessary to explore the model stability on different numbers of missing edges. To achieve it, we randomly kept edges to simulate the situation where tree features cannot be extracted. We retained 90%, 80%, 70%, 60% and 50% of the edges in the image-feature semantic graph and then performed suicide ideation detection on the newly generated graphs, the results are shown in **Figure 9**.

From the results, we can notice that when some edges are removed, the overall performance is relatively stable as the edge size decreases. Besides, macro-F1 and G-mean show a downward trend, and this trend become apparent when the edge size is less than 80%. It can be concluded that the absence of edges does have a slight impact on the performance of suicidal ideation detection, and this impact becomes significant when degree of absence increases to a certain value. Such results indicate that our proposed method is still capable of detecting suicidal ideation when less than 20% characteristics of the tree-drawing image cannot be fully represented. Moreover, such a result once again demonstrates that automatic features extraction should be further studied to improve the performance of the proposed method in the future.

## 4 Discussion

This study employs projective test methodologies, specifically the Tree-Drawing Test (TDT), which effectively uncovers individuals’ subconscious psychological states while minimizing susceptibility to social desirability and subjective control biases. Compared to techniques requiring high-precision equipment and technical expertise, such as electroencephalography, the TDT necessitates only paper and pencil, enabling broader implementation and lower operational costs. This study focuses on how to automatically detect individual suicidal ideation based on the TDT. Beyond machine learning and image processing techniques, we further leverage graph learning to implement automated detection from projective responses. This approach integrates psychological tree features with graph learning techniques, improving the performance of suicidal ideation detection and thereby advancing the application of the TDT in the field of mental health.

Next, it is worth noting that the theoretical basis of TDT is the psychoanalytic genre (48), which heavily relies on experts’ interpretations. To address this issue, existing researches have explored various coding systems to link drawing features with specific mental states (16, 49–52). For instance, trunk width, trunk base opening, and branch ends size are significantly associated with schizophrenia (49). Canopy area, canopy height, canopy width, trunk width and total tree area are related to depressive symptoms (16). Roots, truncated tree, flattened crown, and bizarre tree are considered as the important predictors for mental disorders (50). In our ablation study, “leaves and fruits” demonstrates its effectiveness in detecting suicidal ideation. Generally speaking, in projective tree-drawing test, “leaves and fruits” typically correspond to an individual’s connection with their environment and personal growth and aspirations (53), and thus some researches have adopted “leaves” and “fruit” to predict individual depression (54). This is inherently consistent with our findings. This series of research suggests that projection tests contain rich individual differences, and the relevant features are worth continuously exploring.

Furthermore, considering the associations among negative emotions, abnormal mental state and suicidal ideation, previous studies provide a certain basis for automatic suicidal ideation detection. However, in traditional machine learning methods that initially depend on feature recognition, the accuracy of this feature recognition process significantly impacts model performance. The current study demonstrates that employing graph learning approaches can, to a certain extent, address the issue of performance stability. This may be because graph learning allows for the modeling of complex relationships and interactions between tree-drawing images and tree features (55). This characteristic ensures robust stability of the model under high performance, even when the training set changes. At the same time, the unique “image-feature” semantic graph structure can better explain the inference of the proposed method. Moreover, graph learning models are inherently flexible and adaptable, capable of incorporating new information dynamically. When new image features are added, the graph model can naturally expand by adding new nodes, but traditional machine learning models can only be redesigned and trained (56, 57). Additionally, more and more graph modeling techniques are beginning to focus on the interpretability of model’s decisions (38, 39, 58). Although these complex models did not yield good results on our dataset, they may become future solutions for automated analysis of projection tests as data accumulates.

Finally, there are also some limitations to be explored in the future. First, regarding sample selection, this study only included 806 primary and middle school students from Shaanxi Province, resulting in a small sample size with limited geographic and age diversity. The symbolic meanings of tree drawings may vary across cultural and age groups, so the model’s applicability in different cultural contexts requires further consideration. Second, this study adopted a cross-sectional design, capturing data at a single time point. However, suicidal ideation often exhibits dynamic development, and data from a single time point may be significantly influenced by the testing context and participants’ current psychological states. Future research should consider integrating projective tests into longitudinal designs, capturing the temporal dynamics of suicidal ideation and allowing for a more comprehensive understanding of its progression over time. Next, due to the small data size and the nature of GCN model, this study did not conduct an in-depth analysis of the importance of each tree-drawing image feature. In future research, with the application of advanced graph models supported by a larger dataset, it is expected that we can explain the effectiveness of these features more thoroughly. Finally, the performance of automated recognition of image features in the tree-drawing test still needs improvement, to support more accurate individual suicidal ideation detection.

## Data Availability

The raw data supporting the conclusions of this article will be made available by the authors, without undue reservation.
